# Identification of Allelic Variation in Drought Responsive Dehydrin Gene Based on Sequence Similarity in Chickpea (*Cicer arietinum* L.)

**DOI:** 10.3389/fgene.2020.584527

**Published:** 2020-12-14

**Authors:** Tapan Kumar, Neha Tiwari, Chellapilla Bharadwaj, Ashutosh Sarker, Sneha Priya Reddy Pappula, Sarvjeet Singh, Mohar Singh

**Affiliations:** ^1^Division of Genetics, ICAR-Indian Agricultural Research Institute, New Delhi, India; ^2^International Center for Agricultural Research in the Dry Areas, Bhopal, India; ^3^Department of Plant Breeding & Genetics, Punjab Agricultural University, Ludhiana, India; ^4^Department of Plant Breeding & Genetics, ICAR-NBPGR Regional Station, Shimla, India

**Keywords:** chickpea, drought tolerance, dehydrin (DHN), sequence similarity, morphological characterization

## Abstract

Chickpea (*Cicer arietinum* L.) is an economically important food legume grown in arid and semi-arid regions of the world. Chickpea is cultivated mainly in the rainfed, residual moisture, and restricted irrigation condition. The crop is always prone to drought stress which is resulting in flower drop, unfilled pods, and is a major yield reducer in many parts of the world. The present study elucidates the association between candidate gene and morpho-physiological traits for the screening of drought tolerance in chickpea. Abiotic stress-responsive gene Dehydrin (*DHN*) was identified in some of the chickpea genotypes based on the sequence similarity approach to play a major role in drought tolerance. Analysis of variance revealed a significant effect of drought on relative water content, membrane stability index, plant height, and yield traits. The genotypes Pusa1103, Pusa362, and ICC4958 were found most promising genotypes for drought tolerance as they maintained the higher value of osmotic regulations and yield characters. The results were further supported by a sequence similarity approach for the dehydrin gene when analyzed for the presence of single nucleotide polymorphisms (SNPs) and indels. Homozygous indels and single nucleotide polymorphisms were found after the sequencing in some of the selected genotypes.

## Introduction

Drought is an environmental condition that arises due to the water scarcity and is a result of very low rainfall or water supply. The severity of the drought is determined by its timing and duration (Toker et al., 2007). It is estimated that fifty per cent yield losses are caused by drought and heat stress (Gaur et al., 2012a). Discovering the genotypic variation between the chickpea genotypes for drought tolerance is most important for the execution of breeding programs for chickpea (Kumar et al., 2018). Chickpea (*Cicer arietinum* L.; *Fabaceae* family) is a diploid plant, containing chromosome number (2n = 16), self-pollinated and cool-season pulse. The genome size of chickpea is approx. ~738 Mb and reported to be having an estimated 28,269 genes (Varshney et al., 2013). Chickpea is being grown in more than fifty countries across the globe (Upadhyaya et al., 2011; Gaur et al., 2012b). It is the most important food legume crop, grown in tropical, subtropical, and temperate regions (Mohammed et al., 2017). Chickpea is cultivated mostly in the rainfed condition (Kumar and Abbo, 2001) and drought is a major constraint for chickpea production (Toker et al., 2007).

Chickpea crop responds variably to the drought stress depending upon the variety, growth stage, and stress duration (Maqbool et al., 2017). Considerable variation exists for the morphological and physiological traits for drought resilience at the different developmental stages. Various studies have established several morpho-physiological parameters for the screening of the drought-like days to 50% flowering (DTF), maturity in days (DTM), relative water content (RWC), membrane stability index (MSI), yield components, etc (Bharadwaj et al., 2011; Maqbool et al., 2017; Shah et al., 2020). A rigorous phenotypic screening technique is required for a better understanding of the crop responses under the stress conditions.

Dehydrin (*DHN*) proteins are the stress-responsive proteins observed under low temperature or in dehydration (Hanin et al., 2011). In the total seed, protein dehydrin is present up to 4% and is assumed to be involved in protecting the embryo and seed tissues from osmotic disturbances when available water in the mature seed is very low (Wise and Tunnacliffe, 2004). Transgenic plants overexpressing *DHN* showed better growth and tolerance when exposed to the drought and freezing stress compared to the wild-type plants (Puhakainen et al., 2004a). *DHNs* are one of the many proteins that have been precisely related to qualitative and quantitative changes in the cold hardiness (Close, 1996). It is also found that plants engineered for *DHN* over-expression, displayed better endurance when exposed to the low temperature in the Arabidopsis (Puhakainen et al., 2004b).

Drought stress shortens the growing season, which affects the yield components viz., seed weight, total biomass, pod number, yield per plant, and seed number of the plants (Toker et al., 2007). The unavailability of water negatively affects the various physiological processes associated with crop developments including stomatal regulation, photosynthesis, and transpiration. It also affects cell growth, hormonal, and enzyme concentration (Hsiao, 1973; Boyer and McPherson, 1975; Begg and Turner, 1976). Improved sequencing technology is a quick and low-cost method through which enormous sequence data can be generated and is eventually helpful for the identification of genes responsible for stress tolerance (Castro et al., 2012). Some of the candidate genes known for the abiotic stress tolerance are Snf-1 related kinase (AKIN), DREB2A gene, dehydrin (*DHN*), CAP2 gene, and Myb transcription factor (MYB) (Roorkiwal et al., 2014). The sequences of these abiotic stress-related candidate genes can be used as a reference sequence for crop improvement via molecular breeding, especially for complex traits (Deshmukh et al., 2014). For the present investigation, fifty chickpea genotypes consisting of released varieties, germplasm collections, landraces, and wild derivatives were used for the study of morpho-physiological characters and the sequence similarity identification of *DHN* gene.

## Materials and Methods

Fifty chickpea genotypes consisting of released varieties, germplasm collections, landraces, and wild derivatives were evaluated in two replications under the normal and rainfed conditions at the experimental farm of IARI, NeDelhi (28.6377° N and 77.1571° E) with altitude 228.61 m over mean sea level) during 2015–16 and 2016–17. Field experiments were performed in the randomized block design with two replications for all the genotypes including tolerant and susceptible checks. Genotypes under investigation were grown in two meters and two rows with a spacing of 45 cm between rows and 10 cm within the rows (Supplementary Table 1).

### Morphological Characterization of Chickpea Genotypes by Agronomic Data

Data for the morpho-physiological characters were recorded for days to 50% flowering, maturity in days, hundred seed weight, plant yield, relative water content, and stability index of the membrane. Mean values were used for analysis in CROP-STAT (version 7.2) statistical package: https://cropstat.software.informer.com/7.2/. Pearson's Correlation matrix among the traits under control and rainfed conditions had been generated by employing GenSTAT version 16.1: www.vsni.co.uk/software/ Genstat (Table 1).

**Table 1:** Mean, standard error, coefficient of variation, range, heritability in broad sense, genetic advance and percentage decrease of traits in normal and rainfed condition.

**Traits**	**Normal**					**Drought**					**Percent decrease of traits (%)**
	**Mean ± SE**	**CV (%)**	**Range**	***h*^**2**^**	**GA**	**Mean ± SE**	**CV (%)**	**Range**	***h*^**2**^**	**GA**	
Days to 50% Flowering	78 ± 2.32	20.97	57 −103	0.979	36.518	68 ± 1.79	18.77	49–87	0.984	36.258	13.97
Days to maturity	134 ± 0.32	1.68	124 −136	0.586	4.667	116 ± 0.43	2.61	112–121	0.149	1.073	13.57
Plant height	57 ± 0.97	11.95	44 −75	0.959	15.772	50 ± 0.76	10.76	37–65	0.916	15.405	12.09
Pods per plant	38 ± 1.31	24.49	21 −56	0.718	17.368	24 ± 1.06	30.79	11–44	0.708	17.108	36.63
Seed per pod	2 ± 0.07	30.93	1 −2	0.627	1.699	2 ± 0.07	30.93	1–2	0.488	1.026	0.00
Relative water content	67 ± 1.46	15.48	52 −83	0.808	13.796	66 ± 1.67	17.79	51–90	0.906	18.180	0.35
Membrane stability index	60 ± 1.67	19.60	41 −78	0.921	19.211	59 ± 1.78	21.21	40–80	0.885	16.164	1.08
Yield	79 ± 3.09	27.59	40 −139	0.766	57.805	52 ± 2.44	33.39	24–85	0.737	30.635	34.41

Factorial and clusters analysis for drought based on morpho-physiological traits has been done by using DARwin 5 software 5.0.158 (Perrier and Jacquemoud-Collet, 2006).

#### Relative Water Content (RWC)

Three leaflets on top, middle, and lower part of the plant (0.5 g) were taken for measuring relative water content (%) at 50% podding stage. The calculation was done by the following formula given by Blum and Ebercon (1981).

$$\begin{array}{l} \text{RWC }\left(\text{\%}\right)={\text{F}}_{\text{w}}-{\text{D}}_{\text{w}}/{\text{T}}_{\text{w}}-{\text{D}}_{\text{w}}\text{ x}100 \end{array}$$

Where, F_w_ = Fresh weight, T_w_ = Turgid weight, D_w_ = Dry weight.

#### Membrane Stability Index (MSI)

Two-gram fresh weight of leaf samples were taken to record the membrane stability index at 50% flowering stage. MSI calculations were done by the following formula given by Blum and Ebercon (1981).

$$\begin{array}{l} \text{MSI}=\left(1-{\text{C}}_{1}/{\text{C}}_{2}\right)\text{ x }100 \end{array}$$

Where, C_1_ = Electrical conductivity at 40°C for 30 min

C_2_ = Electrical conductivity at 100°C for 10 min.

### Identification of Candidate Gene Related to Abiotic Stress Tolerance

Based on morpho-physiological characterization data obtained, a subset of genotypes that were found tolerant to drought was selected for the validation of candidate gene linked to drought and for the identification of their allelic variation for the *DHN* gene.

### Gel Extraction of DNA Fragment

The anticipated fragments were cut and scooped out from the gel. Purification of the gel was done as per the recommended protocol by using a gel extraction kit (PureLinkTM Quick gel extraction and PCR purification Combo kit, Invitrogen, Carlsbad, CA). Gel solubilization buffer was added to the gel weight (w/v), which contained DNA, and was dissolved by heating to 50°C for 10 min. The solution containing DNA was loaded to the Pure Link TM Clean-up spin column and a short spin for 1 min at 10,000 RPM was given. Flow-through was discarded and the columns were washed with wash buffer. Purified DNA was eluted with 50 μL of elution buffer and rechecked by electrophoresis and stored at −20°C for further use.

### DNA Sequencing and Sequences Analysis

The PCR amplified products were further confirmed by sequencing. The sequencing of the selected amplicons was determined using an ABI automated sequencer (Chromous Biotech Pvt. Ltd., Bangalore, India).

The raw sequences of the desired candidate gene were aligned by the forward and reverse sequences of each genotype and gene identities were confirmed using BLAST against chickpea reference genome assembly. Similarity searches for the nucleotides were performed using BLAST at NCBI (www.ncbi.nlm.nih). Using the ORF finder, open reading frames (ORFs) were identified in NCBI (http://www.ncbi.nlm.nih.gov/gorf/gorf.html). The DNA sequences were aligned using the BioEdit version 7.2.5. This software was also used to detect single nucleotide polymorphisms (SNPs) and mutation/deletion.

## Results

### Screening of Fifty Chickpea Genotypes for Drought Tolerance Under Irrigated and Rainfed Conditions

**Figure 1:**
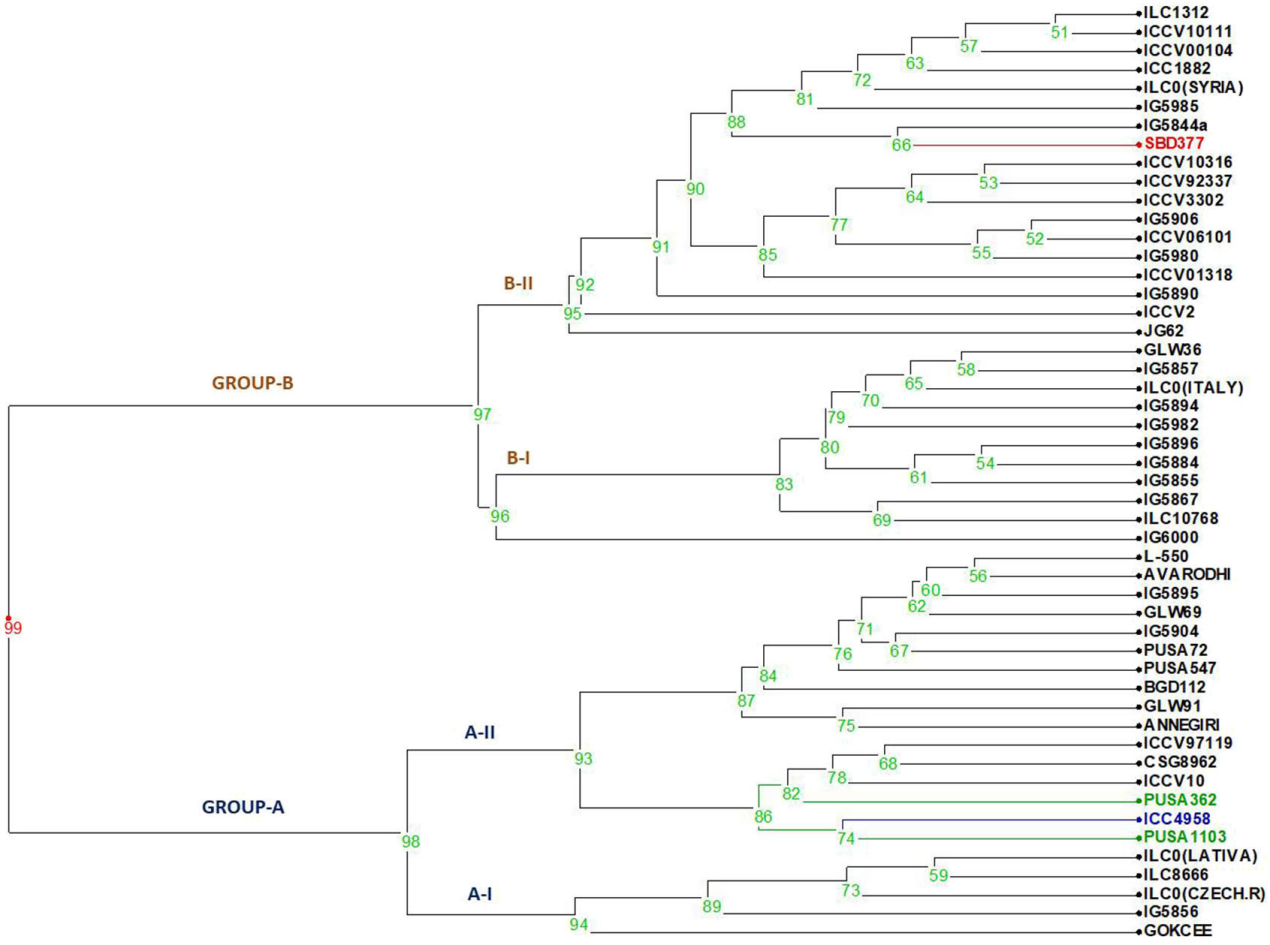
Dendrogram generated from an unweighted pair group method analysis (UPGMA) cluster analysis based on all the stressed morphological characters for drought. The first two clusters form Group A showing all tolerant to moderate tolerant genotypes.

**Figure 2:**
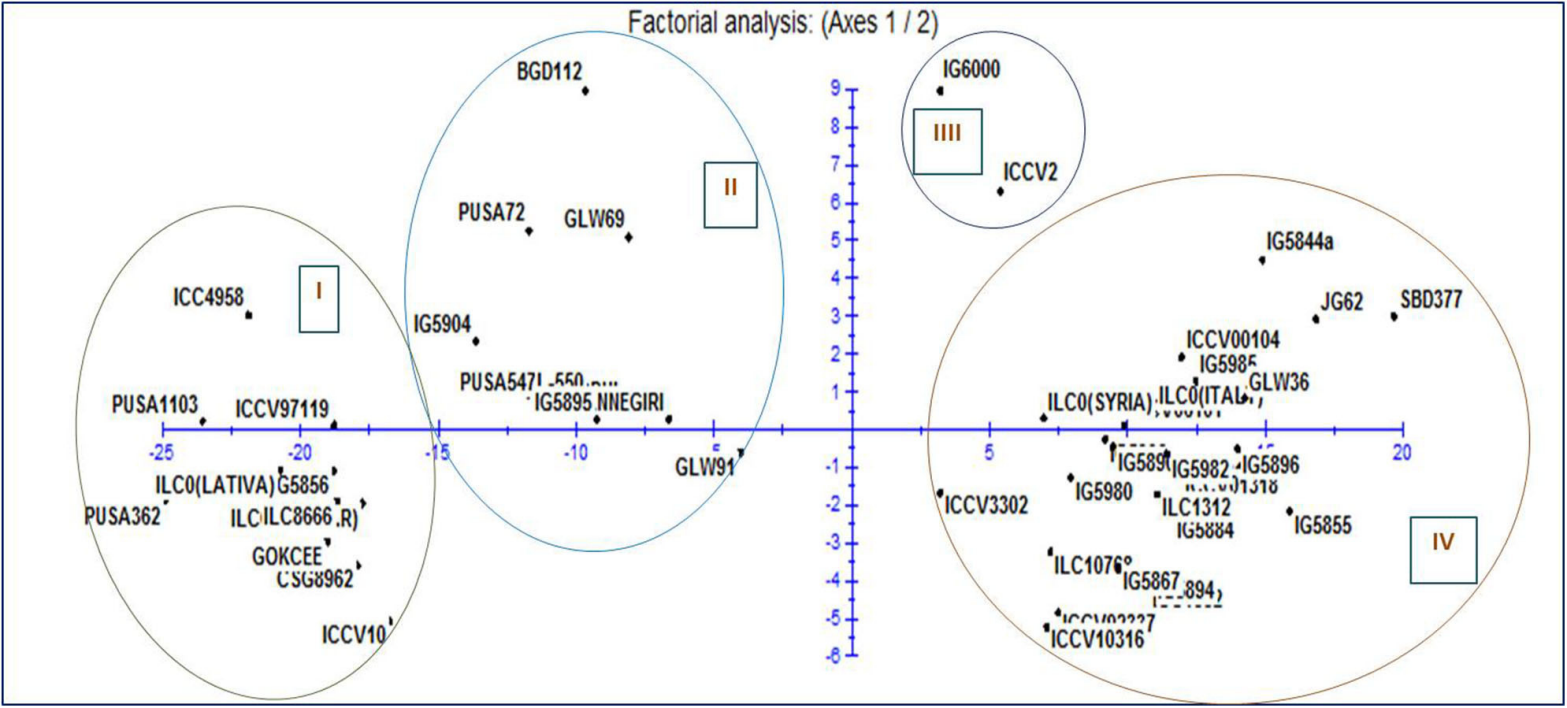
Representation of the 1-2 plane of factorial analysis based on drought stress morphological traits for fifty chickpea genotypes.

Two-way analysis of variance was carried out for all the characters under the normal and rainfed conditions (Table 2). Significant variation was observed under normal and drought environments (Figure 1, Table 3). Days to 50% flowering was positively and significantly correlated with days to maturity (*r* = 0.417). Membrane stability index was negatively correlated with days to flowering (*r* = −0.006). Plant height was positively and significantly correlated with the days to maturity (*r* = 0.47). Pods per plant were positively and significantly correlated with the days to maturity (*r* = 0.602). Relative water content was positively and significantly correlated with the membrane stability index (*r* = 0.912). Seeds per pod was showing a significant but negative correlation with the days to flowering (—0.265). Yield is positively and significantly correlated with days to maturity (*r* = 0.549), membrane stability index (0.585), plant height (0.265) pods per plant (0.588) and relative water content (0.590) (Figure 2, Table 2).

**Table 2:** Two way ANOVA for the morphological traits under normal vs. rainfed conditions.

**Source of variation**	**Mean sum of square**							
	**DTF**	**MSI**	**DTM**	**PH**	**PPP**	**RWC**	**SPP**	**YLD**
**ANOVA table for control vs. drought**								
Genotype	1211.98^**^	878.8^**^	24.694^**^	205.50^**^	289.48^**^	713.7^**^	1.46939	2067.93^**^
Treatment	8769.61^**^	37.45^**^	25208.33^**^	3508.92^**^	13981.01^**^	8.00^**^	0.0	56115.36^**^
Genotype treatment	70.02^**^	15.3^**^	17.81^**^	23.24^**^	138.20^**^	23.3^**^	0.0	258.82^**^
Residual	1.85	0.08	1.874	0.4436	1.672	0.08	0.0	1.599

*, ** *Significance at 5 and 1% respectively; DTF, Days to 50% flowering; MSI, Membrane Stability Index; DTM, Days to Maturity; PH, Plant Height; PPP, Pods per Plant; RWC, Relative Water Content; SPP, Seeds per Pod; YLD, Plant Yield*.

**Table 3:** Pearson's Correlation matrix among the traits under control vs. rainfed conditions.

	**DTF**	**DTM**	**MSI**	**PH**	**PPP**	**RWC**	**SPP**	**YLD**
DTF	1^**^							
DTM	0.417^**^	1^**^						
MSI	– 0.006	0.033	1^**^					
PH	– 0.08	0.47^**^	– 0.075	1^**^				
PPP	0.173	0.602^**^	0.303^**^	0.231^**^	1^**^			
RWC	0.041	0.026	0.912^**^	– 0.076	0.274^**^	1^**^		
SPP	– 0.265^**^	– 0.043	0.16	0.033	0.266^**^	0.227^**^	1^**^	
YLD	0.182	0.549^**^	0.585^**^	0.265^**^	0.588^**^	0.59^**^	0.184	1^**^

*, ** *Significance at 5 and 1% respectively; DTF, Days to 50% Flowering; DTM, Days to Maturity; MSI, Membrane Stability Index; PH, Plant Height; PPP, Pod Per Plant; RWC, Relative Water Content; SPP, Seeds Per Pod; YLD, Plant Yield*.

In the present investigation, high heritability values coupled with high genetic advance were recorded for days to flowering, plant height, pods per plant, relative water content and membrane stability index under irrigated and drought condition. Genotypes retaining early flowering, good plant height, pods per plant, water retention capacity and membrane stability under moisture stress are likely to be more productive under stress environment.

**Figure 3:**
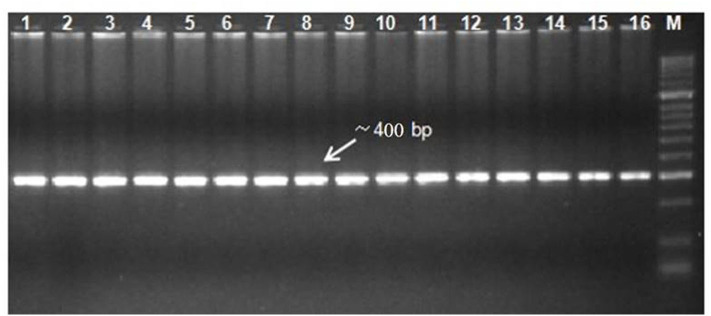
Gel electrophoresis of *DHN* amplicons from different chickpea genotypes (lane: 1-16). Lane M: 2 kb DNA ladder.

**Figure 4:**
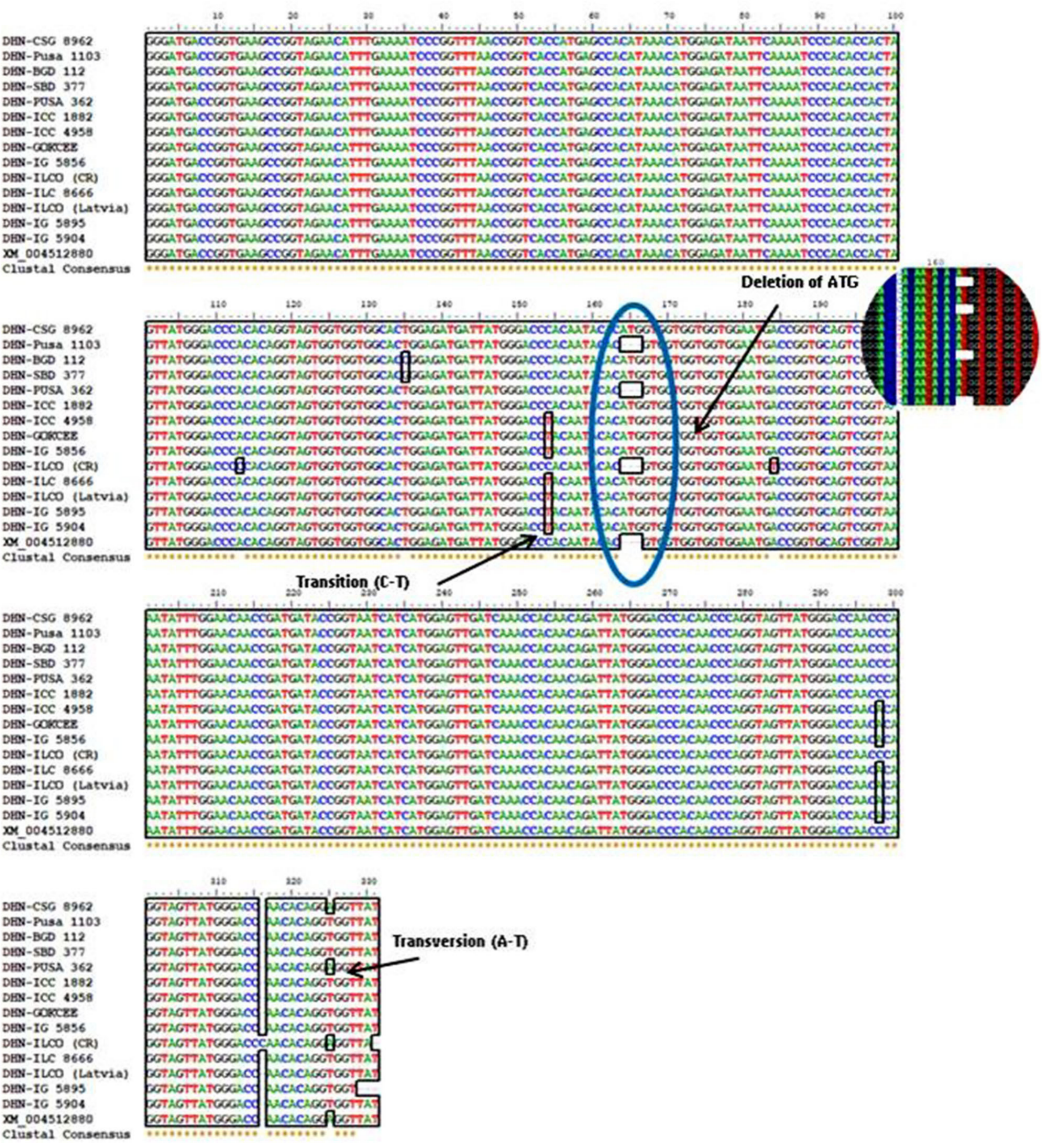
Multiple alignments of nucleotide sequences of the *DHN* gene from different chickpea genotypes were done by using the BioEdit version 7.0.9. The presence of SNP in the *DHN* gene is been indicated.

Moderate heritability was accompanied by low genetic advance for days to maturity and seeds per pods under control and drought condition. The comparison of heritability for all the traits was done under irrigated and drought stress conditions (Table 3). All the morpho-physiological data were analyzed and Euclidean distances were calculated for the stress condition and the genotypes grouped as per their characters. Two distinct groups (A and B) were formed (Figure 3). Tolerant lines were grouped in A and the susceptible lines were in group B. The tolerant genotypes were further divided into two groups, highly tolerant and moderately tolerant. Similarly, the susceptible genotypes were divided into two groups highly susceptible to moderately susceptible. According to all morpho-physiological characters, Pusa1103 and Pusa362 were found the most tolerant genotypes. Also, these tolerant genotypes were grouped with ICC4958 which is a well-known donor for the drought tolerance. A data matrix plot based on the morphological characters had been subjected to Principal Component Analysis (PCoA) for estimating genetic differentiation among the fifty genotypes of chickpea. The scatter plot based on these components disclosed a pattern of mainly two groups. The tolerant genotypes formed a separate group with ICC4958 while the susceptible genotypes formed a group with SBD377. Most of the genotypes were scattered between tolerance and susceptible genotypes. The distribution of genotypes according to geographical origin was lacking in the matrix plot (Figure 4).

### Sequence Similarity and Allelic Variation of the *DHN* Gene

**Figure 5:**
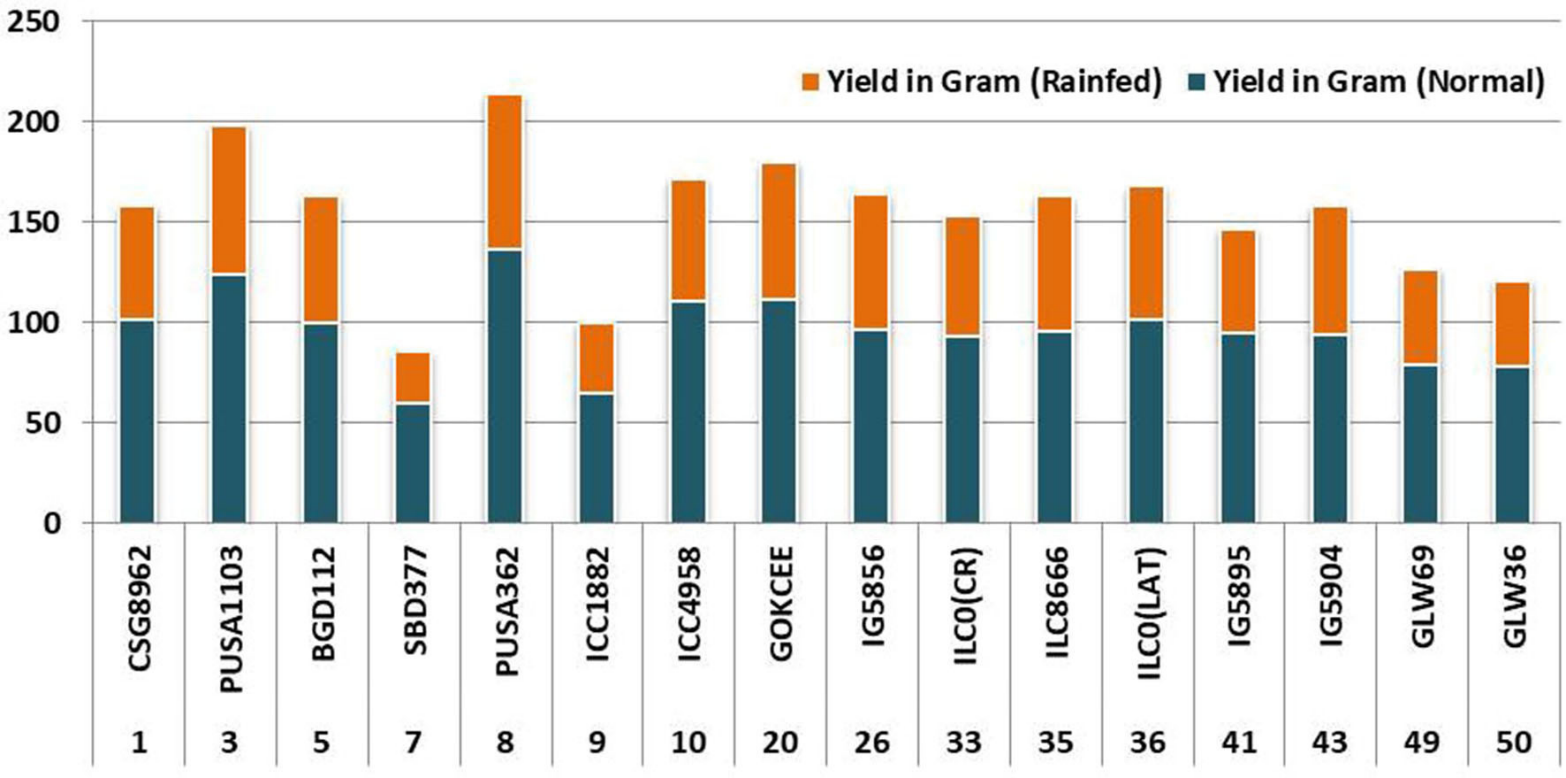
Comparison of the yield of selected genotypes under rainfed and normal conditions.

Based on the morpho-physiological data, a subset of genotypes were selected for the sequence similarity and identification of allelic variation through sequencing. The *DHN* gene sequence was partially amplified through the genomic primer (Roorkiwal and Sharma, 2012). The amplification of the *DHN* gene primer generated a PCR product of ~400 bp in length (Figure 5). The amplified product has been further purified by using a gel purification technique as described in the materials and method section. Purified PCR products were subjected to sequencing (Chromous Biotech Pvt. Ltd., Bangalore, India). Analysis of the sequences and nucleotide identity searches had been done by BLASTN and BLASTX in NCBI (www.ncbi.nlm.nih) and found that the sequences are showing the highest identity with the homologous gene. The nucleotide sequence analysis indicated that the *DHN* gene from different genotypes had diversity among the sequences. The amino acid sequences homology of the *DHN* were found highest with the reference *DHN* gene (XM_004512880).

**Figure 6:**
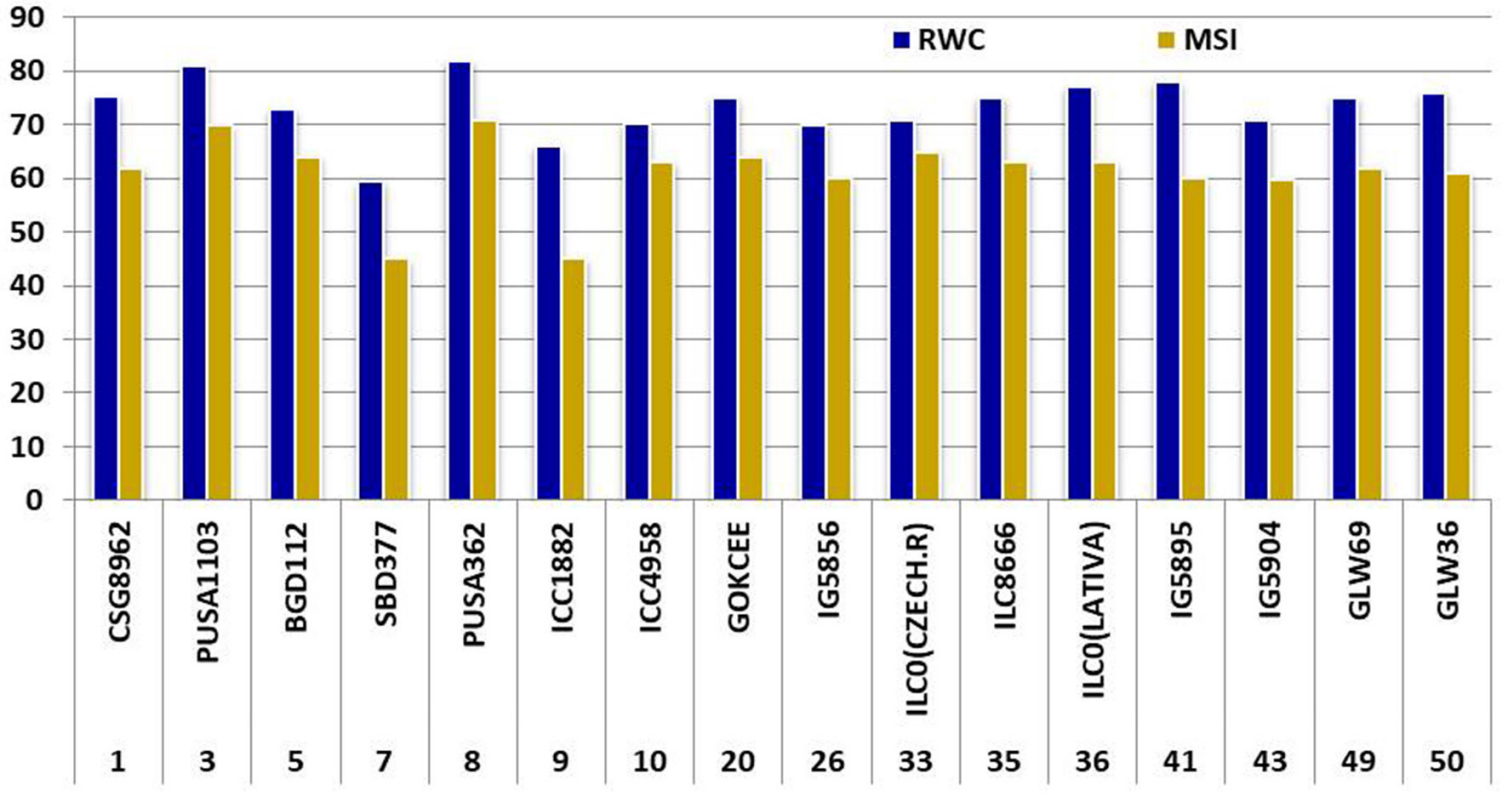
Comparison of the RWC and MSI of selected genotypes.

The sequencing alignment revealed a different number of SNPs in the candidate gene among genotypes. The indels were only present in the candidate gene from genotypes Pusa1103, Pusa362, ILC0 (Latvia), and the reference *DHN* gene (XM_004512880). Also, a single nucleotide polymorphism was present in genotype Pusa362. The base by base comparison revealed that the homozygous alleles of SNPs in the position 154 and 298 are present in the drought-tolerant genotypes (ICC4958, GOKCEE, IG5856, ILC8666, ILC0 (Latvia), IG5895 and IG5904). But, in the case of genotype BGD112 and SBD377 heterozygous alleles of SNPs were found in position 135 (Figure 6, Supplementary Table 2). The results suggest that the genotypes Pusa1103 and Pusa362 having homozygous indels and SNPs showed a significantly higher value of relative water content, membrane stability index, and yield in comparison to other genotypes. The gene sequences were deposited in NCBI with ID's CSG8962 (KY542275), PUSA1103 (MF469826), BGD112 (MF469827), SBD377 (MF469828), PUSA362 (KY542276), ICC1882 (MF469829), ICC4958 (MF469830), GOKCEE (MF469831), IG5856 (MF469832), ILC8666 (MF469833), ILC0 (Latvia) (MF469834), IG5895 (MF469835), IG5904 (MF469836).

## Discussion

The acclimation of plants to drought stress conditions is dynamic and complex, which involves hundreds of genes and their interactions with different environmental factors throughout the plant development (Kumari et al., 2009). Drought has become one of the most important constraints for chickpea production. In recent years, significant improvement by way of breeding for chickpea adaptation to the drought was achieved (Devasirvatham et al., 2012; Kumar et al., 2015). However, a gap still exists in the understanding of the physiological and molecular mechanisms under water stress conditions. It is imperative to study the plant physiological responses under water stress conditions and to develop drought-tolerant chickpea cultivars by utilizing improved screening techniques and various modern genetic approaches. The severity of drought can be estimated by morphological features and physiological processes of plants during its growth and development like days to 50% flowering, days to maturity, plant height, pods per plant, seed per pod, relative water content, membrane stability index, yield (Toker and Cagirgan, 1998; Jaleel et al., 2009). Relative water content and membrane stability index are the best indices that can accurately indicate the balance between water absorbed by the plant and the amount consumed through transpiration. In wheat, Schonfeld et al. (1985) disclosed that the cultivars having high relative water content were more tolerant to drought stress. Ramos et al. (2003) identified significant differences in relative water content in bean leaves and the values were lesser in drought conditions than normal. Many researchers also reported characters like membrane thermostability, canopy temperature depression to be highly effective in screening for drought conditions (Leport et al., 1999).

All the tolerant genotypes had high values of relative water content and membrane stability index. The lower the difference between them, the greater the genotype has tolerance to drought. The genotypes Pusa1103 and Pusa362 not only had lower variation in the relative water content and membrane stability index values but they were also high yielding and thus are promising under normal and rainfed conditions. It is essential to explore the variation for drought indicatory parameters in crops for their effective utilization (Ali et al., 2011 and Dhanda et al., 2004). Relative water content is a function of water uptake by the roots as well as water loss by transpiration and is also considered as a pivotal index for dehydration tolerance. Drought susceptibility is a result of low relative water content in a wide variety of plants including chickpea (Jain and Chattopadhyay, 2010; Yucel and Anlarsal, 2010; Rahbarian et al., 2011). Water stress impairs both membrane structure and function of the plant cells/tissue (Cave, 1981). The cell membrane is one of the first targets of many plant stresses like drought and affects cell membrane integrity and stability (Lyevitt, 1972). Hence, the maintenance of cell membranes integrity and stability under water stress is also one of the measures for tolerance to drought (Vieira da Silva et al., 1974).

Better heritability values of traits were having more possibilities of improvement (Ahmed and Khaliq, 2007; Songsri et al., 2008). High heritability accompanied by low genetic advance for days to maturity and seeds per pods suggested that high heritability may not necessarily lead to increased genetic gain until variability present for the trait (Sardana et al., 2007). It has been accentuated that without hereditary development, the heritability esteems would not be of useful significance in choice dependent on phenotypic appearance. Thus, hereditary development should be considered along with heritability in coherent selection breeding program.

During dehydration conditions, plant cells lose water and membrane-associated complexes and proteins undergo an undesirable denaturation process. Several studies proposed that *DHN* acts as “space-filler” and it gathers relatively in larger amounts in various compartments inside the cells during dehydration (Battaglia et al., 2008). Close (1996) and Allagulova et al. (2003) have shown a positive correlation between the accumulation of *DHN* proteins and tolerance to freezing, drought, and salinity. Thus under dehydration condition dehydrin gene can help in keeping the original cell volume, preventing cellular collapse (Hanin et al., 2011). Various workers also reported the correlated responses of Dehydrin with stress tolerance viz., in chickpea (Gao et al., 2008); oat (Maqbool et al., 2002); rice (Moons et al., 1997) and tobacco (Kim et al., 2005).

*DHN* genes can be explored in developing superior chickpea varieties with improved yield under abiotic stress conditions Roorkiwal and Sharma, 2012. The effect of drought and the response to morpho-physiological changes can be used to identify tolerant genotypes with increased yield (Nam et al., 2001; Martinez et al., 2007). It is important to know the molecular as well as phenotypic responses under the restricted condition and to identify the suitable genotypes that respond in rainfed conditions (Upadhyaya et al., 2012). Knowledge of candidate genes for stress resilience is limiting in chickpea (Lata and Prasad, 2011). Modernization and cost-effectiveness of sequencing technology provide a rapid method for the generation of remarkable sequence data which helps to identify the genes responsible for various stress tolerance. This information would greatly aid in crop improvement through SNPs linked with the preferred trait or directly through a transgenic approach (Ray and Satya, 2011; He et al., 2014).

Sequencing-based allele mining involves PCR-based amplification of alleles of a gene in diverse genotypes, followed by DNA sequencing to identify the nucleotide variance in the alleles. Multiple alleles among the cultivars can be identified through this approach. The method enables us to analyze individuals for haplotype structure and diversity to infer genetic association studies in plants. This allows us to recognize the effect of mutations on gene structure and the location of point mutations or SNPs and insertions or deletions (InDels) to construct haplotypes. Sequencing-based allele mining is found to be an efficient approach to expand the rice blast R gene source and manage damaging blast disease (Vasudevan et al., 2015). The presence of homozygous indels and SNPs in the *DHN* gene in Pusa1103 and Pusa362 genotypes suggests that such changes can be highly associated with drought tolerance response. Furthermore, these genotypes are identified as tolerant as they maintained the higher value of relative water content, membrane stability index, and yield in comparison to other genotypes. This study will help us to identify and characterize the drought-tolerant genotypes by utilizing the morphological traits and allelic variation of the *DHN* gene by sequencing techniques and also to discover the allelic variation of the gene.

Data from the coding regions are regularly in use for the identification of genes responsible for stress tolerance from different plant species like Arabidopsis thaliana, Medicago truncatula, and many more. Identification of allelic variations in the drought-responsive candidate genes from diverse genotypes can provide genomic resources with different alleles to develop improved genotypes for drought tolerance.

The present study provides a comparative study of the candidate gene and morphological traits for drought tolerance in chickpea, which can be used in improving drought tolerance in chickpea.

## Data Availability Statement

The datasets presented in this study can be found in online repositories. The names of the repository/repositories and accession number(s) can be found at: https://www.ncbi.nlm.nih.gov/, KY542276; https://www.ncbi.nlm.nih.gov/, MF469826; https://www.ncbi.nlm.nih.gov/, MF469830; https://www.ncbi.nlm.nih.gov/, MF469827; https://www.ncbi.nlm.nih.gov/, KY542275; https://www.ncbi.nlm.nih.gov/, MF469828; https://www.ncbi.nlm.nih.gov/, MF469829; https://www.ncbi.nlm.nih.gov/, MF469831; https://www.ncbi.nlm.nih.gov/, MF469832; https://www.ncbi.nlm.nih.gov/, MF469833; https://www.ncbi.nlm.nih.gov/, MF469834; https://www.ncbi.nlm.nih.gov/, MF469835; https://www.ncbi.nlm.nih.gov/, MF469836.

## Author Contributions

TK contributed to the analysis, interpretation of data, prepared the manuscript, and conceived the study. NT contributed to the analysis and interpretation of data. CB designed the study and reviewed and edited the manuscript. AS provided feedback and edited the manuscript. SP contributed to the analysis and interpretation of data. SS reviewed and edited the manuscript. MS provided guidance and edited the manuscript. All the authors read and approved the manuscript.

## Conflict of Interest

The authors declare that the research was conducted in the absence of any commercial or financial relationships that could be construed as a potential conflict of interest.
