# Supplementary material for: Identification of Allelic Variation in Drought Responsive Dehydrin Gene Based on Sequence Similarity in Chickpea (Cicer arietinum L.)
Source: Front Genet. 2020 Dec 14;11:584527. doi: 10.3389/fgene.2020.584527 (PMC7767992; doi:10.3389/fgene.2020.584527)
Supplement: Supplementary file 1 [file Table_1.docx]

**Supplementary Table 1.** List of the fifty chickpea genotypes used under the investigation.

| **S. No.** | **Variety** | **Type** | **Pedigree/Source** |
| --- | --- | --- | --- |
| 1 | CSG8962 | Desi | Selection from GPF 7035 |
| 2 | JG62 | Desi | Local selection from west Nimar (M.P.) |
| 3 | PUSA1103 | Desi | (Pusa256 x C.reticulatum) x Pusa 362 |
| 4 | AVARODHI | Desi | (T-3 x K 315) |
| 5 | BGD112 | Desi Green | (BG 209 x GL 84038) x Pusa 212 |
| 6 | ANNEGIRI | Desi | Local selection from germplasm of Karanataka |
| 7 | SBD377 | Desi | ICCV 88109 x PRR 1) x ICC 4958 |
| 8 | PUSA362 | Desi | (BG 203 x P 179) x BC 203 |
| 9 | ICC1882 | Desi | Traditional landrace P1506-4 from ICRISAT |
| 10 | ICC4958 | Desi | GW 5/7, a drought-tolerant breeding line from ICRISAT |
| 11 | PUSA547 | Desi | Mutant of BG 256 |
| 12 | PUSA72 | Desi | (Pusa 256 x E 100Ym) x (Pusa 256) |
| 13 | ICCV97119 | Desi | (avarodhixJG 62)X ICC 11551 |
| 14 | ICCV06101 | Desi | ICC 5619 x ICC 37 |
| 15 | ICCV00104 | Desi | JG 74 x ICCL 83105 |
| 16 | ICCV10111 | Desi | ICCV 93954 x ICC5683 |
| 17 | L550 | Kabuli | Pb7 x Rabat |
| 18 | ICCV10 | Desi | P1231 × P1265 |
| 19 | ICCV2 | Kabuli | F3 [(K 850 x GW 5/7) x P 458] x F3  (L 550x Guamuchil)-2 |
| 20 | GOKCEE | Kabuli | (Pusa 256 x ICCV 32) x ICCV 32 |
| 21 | ICC 92337 | Kabuli | (ICCV 2x surutato 77) X ICC 7344 |
| 22 | ICCV10316 | Kabuli | ICCV 92337 X ICC 17109 |
| 23 | ICCV03302 | Kabuli | (L550 X ICC 14196)X ICCV 92392 |
| 24 | ICCV01318 | Kabuli | ICCV 95311 X ICC 14194 |
| 25 | IG5844a | Kabuli | Chickpea Landrace from Amman, Jordan |
| 26 | IG5856 | Kabuli | Chickpea Landrace from Maan, Jordan |
| 27 | IG5857 | Kabuli | Chickpea Landrace from Maan, Jordan |
| 28 | IG5884 | Kabuli | Chickpea Landrace from Ninawa, Iraq |
| 29 | IG5894 | Kabuli | Chickpea Landrace from Arbil, Iraq |
| 30 | IG5906 | Kabuli | Chickpea Landrace from As Sulaymaniyah, Iraq |
| 31 | ILC0 (Italy) | Kabuli | Chickpea Landrace from Italy |
| 32 | ILC10768 | Desi | Chickpea Landrace from Armenia |
| 33 | ILC0 (Czech Rep.) | Desi | Chickpea Landrace from the Czech Republic |
| 34 | ILC0 (Syria) | Desi | Chickpea Landrace from Icarda Syria |
| 35 | ILC8666 | Kabuli | Chickpea Landrace from Portugal |
| 36 | ILC0(Latvia) | Desi | Chickpea Landrace from Latvia |
| 37 | ILC1312 | Kabuli | Chickpea Landrace from Cyprus |
| 38 | IG5855 | Kabuli | Chickpea Landrace from Jordan |
| 39 | IG5867 | Kabuli | Chickpea Landrace from Jordan |
| 40 | IG5890 | Kabuli | Chickpea Landrace from Iraq |
| 41 | IG5895 | Kabuli | Chickpea Landrace from Iraq |
| 42 | IG5896 | Kabuli | Chickpea Landrace from Iraq |
| 43 | IG5904 | Kabuli | Chickpea Landrace from Iraq |
| 44 | IG5980 | Kabuli | Chickpea Landrace from Esp |
| 45 | IG5982 | Kabuli | Chickpea Landrace from Esp |
| 46 | IG5985 | Kabuli | Chickpea Landrace from Esp |
| 47 | IG6000 | Kabuli | Chickpea Landrace from Tunisia |
| 48 | GLW91 | Desi | A breeding line from PAU resistant to Aschochyta Blight. |
| 49 | GLW69 | Desi | A breeding line from PAU resistant to BGM |
| 50 | GLW36 | Desi | A breeding line from PAU resistant to BGM |
