# Supplementary material for: Identification of Allelic Variation in Drought Responsive Dehydrin Gene Based on Sequence Similarity in Chickpea (Cicer arietinum L.)
Source: Front Genet. 2020 Dec 14;11:584527. doi: 10.3389/fgene.2020.584527 (PMC7767992; doi:10.3389/fgene.2020.584527)
Supplement: Supplementary file 2 [file Table_2.docx]

**Supplementary Table 2.** List of SNPs/InDels were categorized as transition, transversion and indels in the DHN sequences.

| **Nucleotide Substitution** | **Genotype** | **Base No.** | **Sequence** | **Frequency** |
| --- | --- | --- | --- | --- |
| InDels | Pusa 1103 | 182 | ATG | 1 |
|  | Pusa 362 |  |  |  |
|  | ILCO (CR) |  |  |  |
|  | GLW 69 |  |  |  |
|  | GLW 36 |  |  |  |
|  | XM_004512880 |  |  |  |
| SNPs (Transitions) | BGD 112 | 153 | T/C | 1 |
|  | ICC 4958 | 172 | C/T | 1 |
|  | GOKCEE |  |  |  |
|  | IG 5856 |  |  |  |
|  | ILC 8666 |  |  |  |
|  | ILCO (Lativa) |  |  |  |
|  | IG 5895 |  |  |  |
|  | IG 5904 |  |  |  |
| SNPs (Transversions) | SBD 377 | 153 | T/G | 1 |
|  | ILCO (CR) | 131 | A/C | 1 |
